# Venetoclax efficacy on acute myeloid leukemia is enhanced by the combination with butyrate

**DOI:** 10.1038/s41598-024-55286-0

**Published:** 2024-02-29

**Authors:** Renshi Kawakatsu, Kenjiro Tadagaki, Kenta Yamasaki, Tatsushi Yoshida

**Affiliations:** Department of Biochemistry and Molecular Biology, Graduate School of Medical Science, Kyoto Prefectural University of Medicine, Kawaramachi-Hirokoji, Kamigyo-ku, Kyoto, 602-8566 Japan

**Keywords:** Cancer, Haematological cancer, Leukaemia

## Abstract

Venetoclax has been approved recently for treatment of Acute myeloid leukemia (AML). Venetoclax is a BH3-mimetic and induces apoptosis via Bcl-2 inhibition. However, venetoclax’s effect is still restrictive and a novel strategy is needed. In the present study, we demonstrate that sodium butyrate (NaB) facilitates the venetoclax’s efficacy of cell death in AML cells. As a single agent, NaB or venetoclax exerted just a weak effect on cell death induction for AML cell line KG-1. The combination with NaB and venetoclax drastically induced cell death. NaB upregulated pro-apoptotic factors, Bax and Bak, indicating the synergistic effect by the collaboration with Bcl-2 inhibition by venetoclax. The combined treatment with NaB and venetoclax strongly cleaved a caspase substrate poly (ADP-ribose) polymerase (PARP) and a potent pan-caspase inhibitor Q-VD-OPh almost completely blocked the cell death induced by the combination, meaning that the combination mainly induced apoptosis. The combination with NaB and venetoclax also strongly induced cell death in another AML cell line SKNO-1 but did not affect chronic myeloid leukemia (CML) cell line K562, indicating that the effect was specific for AML cells. Our results provide a novel strategy to strengthen the effect of venetoclax for AML treatment.

## Introduction

Acute myeloid leukemia (AML) is a hematological malignancy and the most common type of adult leukemia^1–3^. AML possesses characteristics of high heterogeneity and genetic diversity with gene mutations^4,5^. AML has high mortality rate and the 5-year overall survival rate is still less than 50%^6,7^. Moreover, most of AML patients frequently become relapse and chemotherapy resistance^8–10^. Thus, new treatment strategies of AML are needed.

As a useful strategy for the treatment of malignant tumors, cell death induction by means of chemotherapy is promising and many anti-tumor agents have been developed to target molecules related to cell death^11–13^. Three major types of cell death, apoptosis (type I), autophagic cell death (type II) and necrotic cell death (type III)^14,15^, and some novel types of cell death such as necroptosis^13,16^, ferroptosis^17^ and pyroptosis^18^ have been reported. Among the cell death, apoptosis has been well analyzed in terms of its mechanism^19–21^. Apoptosis is induced by an increase the mitochondrial outer membrane permeability (MOMP). The change in MOMP acts as a trigger of cytochrome C release from mitochondria into the cytoplasm and then, cysteine aspartate-specific proteases (caspases) are activated and cleave numerous substrates, resulting in apoptosis. The increase of MOMP during apoptosis execution is regulated by the B cell leukemia gene-2 (Bcl-2) family antagonistically^22^. Pro-apoptotic factors such as Bcl-2-associated X protein (Bax) and Bcl-2 homologous antagonist/killer (Bak) are considered to multimerize in response to apoptotic stimuli and forms pore on mitochondria causing an increase of MOMP. On the other hand, Bcl-2 and Bcl-XL interact with Bax or Bak via thoseBH3-domain to inhibit multimerization and act as anti-apoptotic factors. Thus, inhibition of Bcl-2 and Bcl-XL can promote apoptosis in malignant tumor cells.

A small molecule mimetic of the BH3 domain, navitoclax (ABT-263) was first developed as an anti-tumor agent to induce apoptosis in malignant tumor cells^23,24^. Nabitolax broadly binds to anti-apoptotic Bcl-2 family such as Bcl-2, Bcl-XL and Bcl-w. Navitoclax showed good effect on chronic lymphocytic leukemia (CLL) and small-cell lung cancer in clinical trials, however, it showed dose-limiting thrombocytopenia because of the role of Bcl-XL in platelet production^25,26^. Consequently, venetoclax (ABT-199), a selective inhibitor of Bcl-2 was developed to reduce the thrombocytopenic effect^27^. Venetoclax was tested in phase III clinical trials for CLL in combination with rituximab and the progression-free period increased to 57.3% in venetoclax + rituximab against 4.6% in only rituximab and the overall survival also increased to 85.3% in venetoclax + rituximab against 66.8% in only rituximab after 4 years^28^. The U. S. Food and Drug administration (FDA) approved venetoclax for patients with relapsed or refractory CLL in 2016. In the clinical study of AML, phase III trials have been conducted in combination with azacitidine or low-dose cytarabine (LDAC), and in both cases, median overall survival has been prolonged (14.7 months in azacytidine + venetoclax group vs. 9.6 months in azacytidine alone group and 7.2 months in venetoclax + LDAC group vs. 4.1 months in LDAC alone group)^29,30^. The FDA also approved venetoclax for patients age 75 and older with newly diagnosed AML or who have complications that made intensive induction chemotherapy ineligible in 2018. The effects of venetoclax treatment with azacitidine or cytarabine are still restrictive and new combination treatments have been developed^31–33^. Therefore, new strategies to strengthen the venetoclax’s efficacy are urgently required.

Sodium butyrate (NaB) is a short chain fatty acid produced by a metabolism of dietary fibers. NaB possesses a property to inhibit cell growth in malignant tumors^34–38^ and the effects are considered to be mediated by the function as a HDAC inhibitor^39,40^. We previously reported that NaB sensitized various human malignant tumors to anti-tumor cytokine, tumor-necrosis factor related apoptosis ligand (TRAIL)^41–43^ through the upregulation of its receptor^44^. Moreover, The combination of NaB with TRAIL effectively killed t(8;21) AML cells in which TRAIL was downregulated by a RUNX1-ETO fusion protein^45^. Considering the tumoricidal effect of NaB, we hypothesized that NaB could overcome the restrictive efficacy of venetoclax in AML treatment.

The present study here demonstrates that NaB enhances venetoclax’s efficacy against AML cells through an increase of apoptosis induction.

## Materials and methods

### Cell culture

In this experiment, we used three cell lines. Acute myeloid leukemia (AML) cell line KG-1 was obtained from Riken BioResource Center and cultivated in RPMI-1640 (FUJIFILM Wako Pure Chemical Corporation, Osaka, Japan) with 10% Fatal bovine serum (FBS) (Sigma-Aldrich; Merck KGaA, Burlington, MA). AML cell line SKNO-1 was obtained from the JCRB cell bank and cultivated in RPMI-1640 with 10% FBS and 10 ng/ml recombinant human granulocyte–macrophage colony-stimulating factor (GM–CSF) (Pepro Tech, Rocky Hill, NJ). Chronic myeloid leukemia (CML) cell line K562 was obtained from Riken BioResource Center and cultivated in RPMI-1640 with 10% FBS. We maintained these cells at 37 ℃ in humidified air with 5% CO_2_.

### Reagents

We obtained Venetoclax (also called ABT-199) from Selleckchem (Houston, TX). We purchased Pan-caspase inhibitor Q-VD-OPh from MedChemExpress (Monmouth Junction, NJ) and added to cells at 20 μM. We obtained Sodium butyrate from Wako (FUJIFILM Wako Pure Chemical, Osaka, Japan) and made use of it in this experiment as NaB. We dissolved all reagents into a solvent Dimethyl sulfoxide (DMSO).

### Western blotting

We lysed cells in RIPA buffer which was contained aprotinin. The cells suspended in RIPA buffer was centrifuged to eliminate the pellet from the supernatant. We collected the supernatant as cell lysate. It was dissolved in 7.5% sodium dodecyl sulfate–polyacrylamide gel (SDS–PAGE) and proteins were transplanted to a nitrocellulose filter. The filter was incubated with poly (ADP-ribose) polymerase (PARP) antibody (#9542) (Cell signaling technology, Danvers, MA) or β-actin antibody (PM053) (Medical and Biological Laboratories, Tokyo, Japan). Then, we recognized the signal by ECL Western blotting detection reagents (GE healthcare, Chicago, IL) and image quant LAS500 (GE healthcare, Chicago, IL).

### RT-qPCR

We extracted total RNA from cells using Isogen II reagent (Nippongene, Tokyo, Japan). Equal amount of total RNA was reverse-transcribed to cDNA by superscript IV reverse transcriptase (Thermo Fisher scientific, Waltham, MA). We generated the cDNA from total RNA with Thunderbird SYBR qPCR mix (Toyobo Life Science, Osaka, Japan), StepOne plus real-time PCR system (Thermo Fisher scientific) and specific primers following.

Human BAX (Forward; 5′-TCAGGATGCGTCCACCAAGAAG, reverse; 5′-TGTGTCCACGGCGGCAATCATC), human BAK1 (Forward; 5′-ATGGTCACCTTACCTCTGCAA, reverse; 5′-TCATAGCGTCGGTTGATGTCG), human Bcl-2 (Forward; 5′-TGGGATGCCTTTGTGGAACTGTA, reverse; 5′-ATATTTGTTTGGGGCAGGCATGT), human Actin (Forward; 5′-GCTGTGCTACGTCGCCCTG, reverse; 5′-GGAGGAGCTGGAAGCAGCC).

### Cell counts

First, we mixed cultured-cell solution with the same amount of trypan blue solution. Next, we observed cells by using Burker-Turk counting chamber (Erma, Saitama, Japan) under an Olympus CK40 inverted light microscope (Olympus, Tokyo, Japan). We regarded cells stained blue by trypan blue as dead cells because dead cells could not emit trypan blue dye.

### Flow cytometry analysis

We obtained cells by centrifugation and suspended the cell pellet in propidium iodide (PI) solution. The propidium iodide solution containing PBS, 0.1% triton- × 100 and 10 microgram/ml of propidium iodide. By making use of BD FACS Canto II and BD FACS diva (BD Biosciences, Franklin Lakes, NJ), we analyzed cells stained with PI for DNA contents.

### Statistical analysis

We used a two-tailed, unpaired Student’s t-test in order to analyze the data obtained from this experiment. We used Microsoft Excel (Microsoft, Redmond, WA) for the analyses.

## Results

### Venetoclax just weakly inhibited cell growth of AML cells

**Figure 1:**
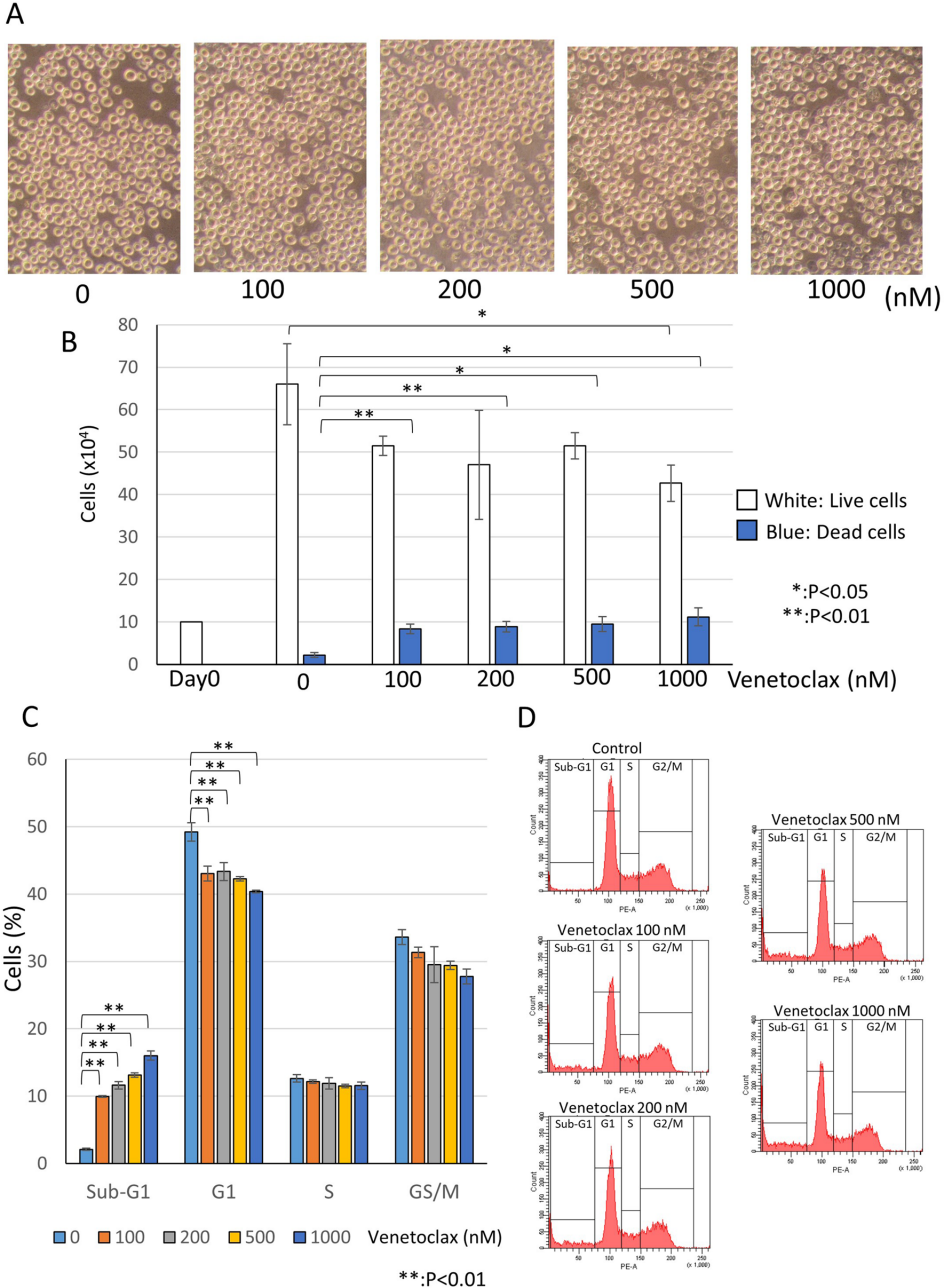
Venetoclax rarely affected for cell growth of AML cells. KG-1 cells which were derived from AML patient were treated with venetoclax for 48 h at the indicated concentrations. (**A**) Cell morphologies were observed using a microscope. (**B**) Trypan blue dye exclusion assay to examine the rate of live or dead cells. (**C**,**D**) Cell cycle analyses of cells stained with PI using flow cytometry. A bar graph with triplicate data was shown in (**C**). Representative histograms were shown in (**D**). The data was n = 3 ± S.D.

We investigated the effect of venetoclax, which is clinically used for AML treatment, on AML cell line KG-1cells. Venetoclax did not markedly affect cell morphology and numbers (Fig. 1A). Next, we examined cell growth using a trypan blue dye exclusion assay. In this assay, live cells can exclude the dye out of cells and are not stained in blue. On the other hand, dead cells cannot exclude the dye and are stained blue. After treatment of venetoclax in KG-1 cells, we counted the number of white or blue cells. As shown in Fig. 1B, dead cells stained blue only weakly increased with dose-dependency. Then, we performed cell cycle analysis using flow cytometry (Fig. 1C,D). Sub-G1 population represents dead cells because dead cells have lower DNA contents by DNA degradation than that of cells at G1 phase. Venetoclax treatment weakly increased sub-G1 population, which was consistent with the result of the trypan blue assay. Other cell cycle populations, G1, S and G2/M were not changed. These results indicated that only venetoclax treatment did not affect markedly for cell growth and cell death of KG-1 AML cells even at high concentration.

### Combination effect of venetoclax with sodium butyrate (NaB)

**Figure 2:**
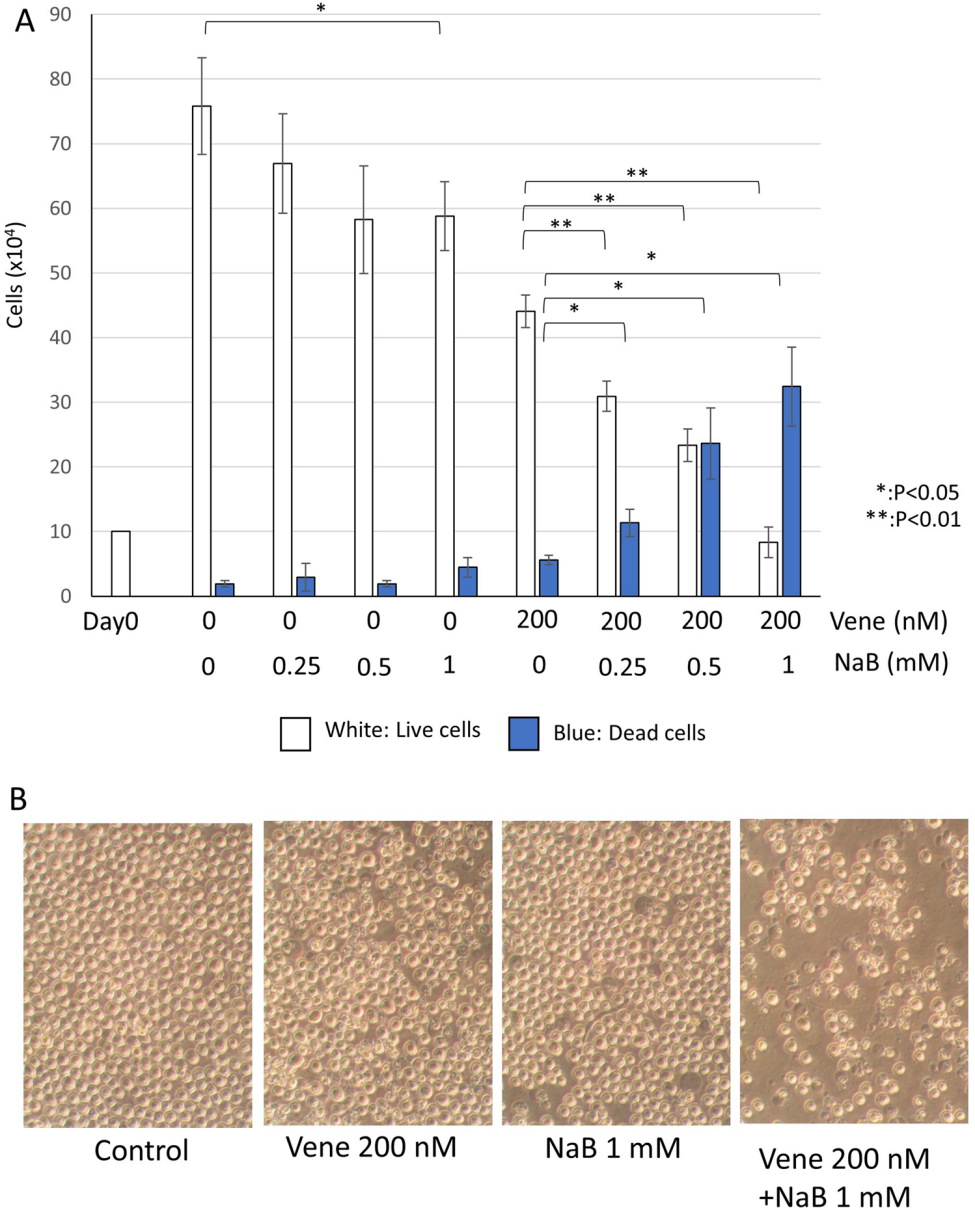
Sodium butyrate (NaB) enhanced venetoclax’s effect on cell growth inhibition. KG-1 cells were treated with venetoclax with or without NaB for 48 h at the indicated concentrations. (**A**) Trypan blue dye exclusion assay to examine the rate of live or dead cells. (**B**) Cell morphologies were observed using a microscope. The data was n = 3 ± S.D.. Vene; venetoclax.

**Figure 3:**
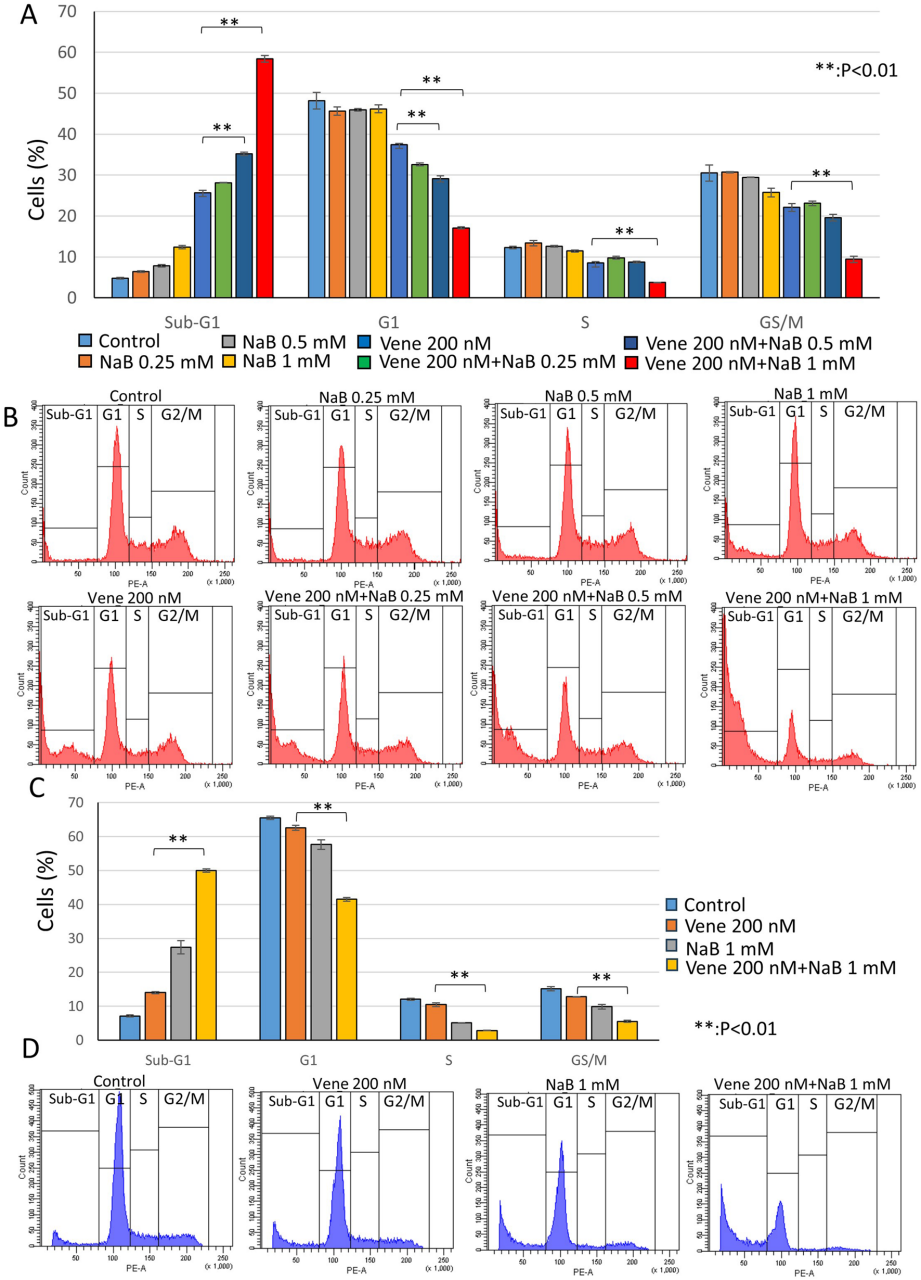
The combined treatment of venetoclax with NaB drastically induced cell death. AML cells (**A**,**B** KG-1 cells; **C**,**D** SKNO-1 cells) were treated with venetoclax with or without NaB for 48 h at the indicated concentrations. Cell cycle analyses of cells stained with PI using flow cytometry. Bar graphs (**A**,**C**) and representative histograms (**B**,**D**) were shown. The data was n = 3 ± S.D.. Vene; venetoclax.

NaB is a short chain fatty acid derived from metabolism of dietary fibers. We previously reported that NaB possessed the ability to enhance tumoricidal effect of TRAIL^44,45^. Thus, we investigated the effect of NaB on anti-tumor efficacy of venetoclax. First, we examined whether NaB inhibited cell growth of KG-1 cells as single agent. As shown in Fig. 2A, only NaB treatment weakly decreased the cell number of KG-1 cells but dead cells were not markedly increased like the effect of venetoclax. The combination of venetoclax with NaB drastically reduced live cells and increased dead cells. The treatment with 200 nM venetoclax and 1 mM NaB killed most part of cells. The observation under a microscope also showed that the cell morphology treated with a single agent of venetoclax or NaB was similar to that of control, however the combination with two agents decreased the cell number and made cells shrunken or dark color, indicating dead cells (Fig. 2B). Next, we performed cell cycle assay with a flow cytometer to investigate the rate of the cell cycle population and dead cells (Fig. 3A,B). Only NaB treatment did not change cell cycle population, though sub-G1 slightly increased. Only venetoclax treatment also slightly increased sub-G1. The combination with NaB and venetoclax drastically increased sub-G1 in a NaB-dose dependent manner. The treatment with 200 nM venetoclax and 1 mM NaB caused about 60% of sub-G1. Other cell cycle populations were reduced along with sub-G1 increase. We used another AML cell line SKNO-1, containing t(8;21) chromosome translocation with RUNX1-ETO fusion protein expression. In SKNO-1, single agent of venetoclax or NaB also had mild effect on sub-G1, however about a half of cells died by the combined treatment (Fig. 3C,D). These results show the combination with NaB enhances the venetoclax’s cytotoxic effect.

### NaB upregulated Bax and Bak expressions

**Figure 4:**
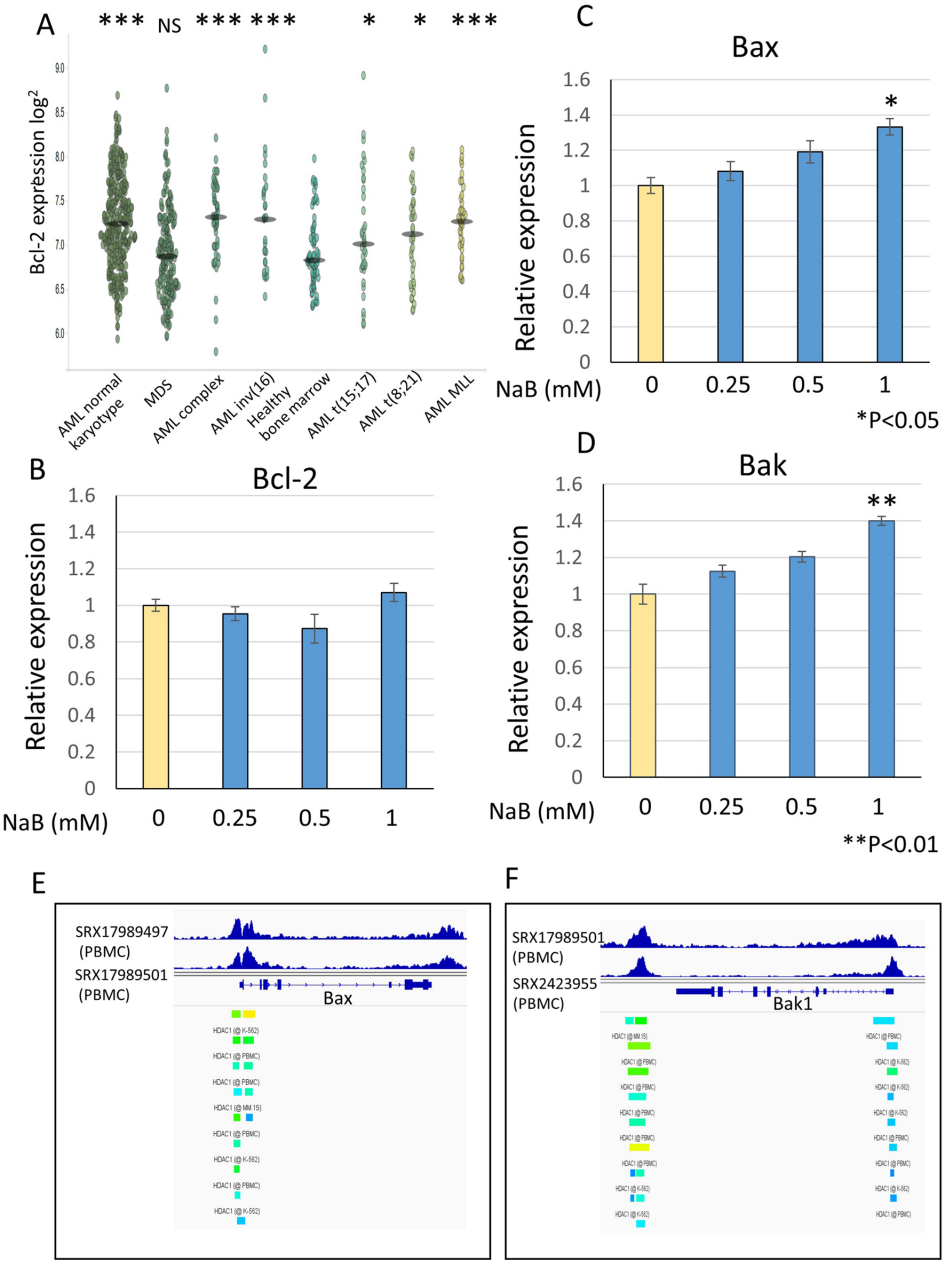
NaB upregulated Bax and Bak expressions. (**A**) BloodSpot analysis of Bcl-2 expression in AML patients. *P < 0.05, ***P < 0.001 and NS = non significant. (**B**–**D**). KG-1 cells were treated with NaB at the indicated concentrations and total RNA was extracted from cells. Bcl-2 (**B**), Bax (**C**) and Bak (**D**) expressions were examined by RT-qPCR with specific primers. The data was standardized by Actin expression. The data was n = 3 ± S.D.. (**E**) and (**F**). ChIP-Atlas analysis of HDAC1 on Bax (**E**) and Bak (**F**) gene locus. ChIP data were derived from SRX17989497, SRX17989501, SRX17989501 and SRX2423955.

To reveal the mechanism of the combined effect of venetoclax with NaB, expression of Bcl-2 family was examined. First, we analyzed Bcl-2 expression via public data BloodSpot (www.bloodspot.eu/)^46^. All types of AML but not myelodysplastic syndromes (MDS) significantly upregulated Bcl-2 compared with healthy bone marrow (Fig. 4A). Next, we examined Bcl-2 expression by RT-qPCR. Bcl-2 expression did not change by NaB treatment (Fig. 4B). On the other hand, pro-apoptotic Bcl-2 family members, Bax and Bak expressions were increased in a NaB’s dose dependent manner (Fig. 4C,D). Venetoclax treatment alone did not affect for Bax and Bak expressions (Supplemental Fig. 1). NaB possesses a property of HDAC inhibition and increases transcription of target genes through histone acetylation. We examined whether Bax and Bak expression were attenuated by HDAC. Using public data ChIP-Atlas^47^, HDAC1 existed on Bax and Bak gene locus in hematopoietic cells (Fig. 4E,F). Analysis of public ChIP-seq data showed that patterns of histone acetylation were changed by butyrate treatment in HDAC1-binding regions and the flanking regions, on Bax and Bak gene locus (Supplemental Fig. 2). These results indicated that NaB inhibited HDAC activity on Bax and Bak genes, leading to activate transcription of Bax and Bak.

### Caspases activation was induced by the combination of venetoclax and NaB

**Figure 5:**
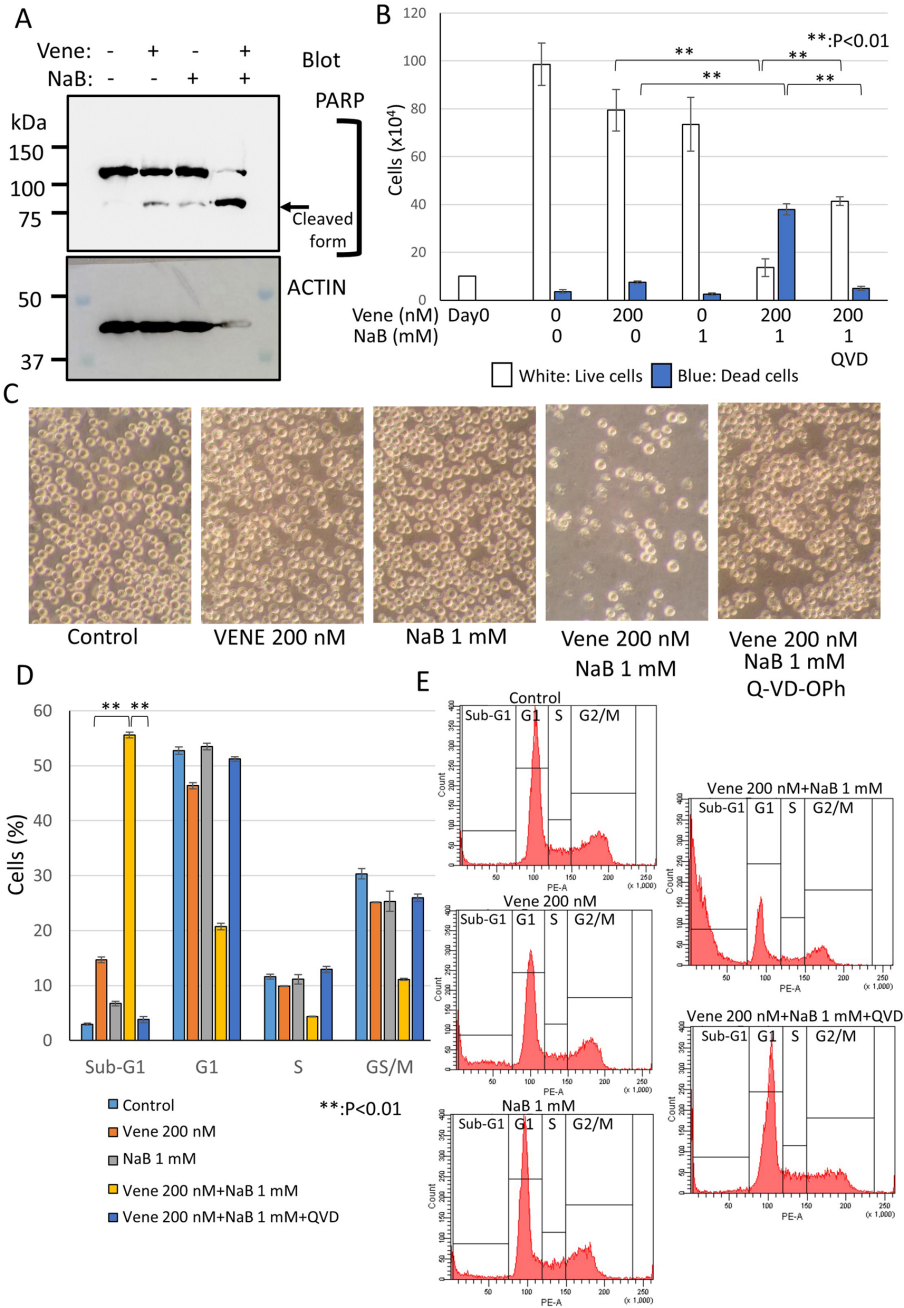
Caspase activation was induced by the combination of venetoclax and NaB. KG-1 was treated with venetoclax alone or the combination with venetoclax and NaB for 48 h. (**A**) Western blotting of PARP. Cell lysates were extracted and western blotting was performed. Upper panel; PARP, Lower panel; actin. Molecular weight marker was shown at the left side. The PARP-cleaved form generated by caspase activation was indicated by an arrow. Original blots are presented in Supplemental Fig. 3. Quantification of band intensity was shown in Supplemental Fig. 4. (**B**) Trypan blue dye exclusion assay to examine the rate of live or dead cells. (**C**) Cell morphologies were observed using a microscope. (**D**,**E**) Cell cycle analyses of cells stained with PI using a flow cytometry. A bar graph with triplicate data (**D**) and representative histograms (**E**) were shown. The data was n = 3 ± S.D.. Vene; venetoclax.

We confirmed the effect of cell death induction by the combined treatment using western blotting of a caspase substrate poly (ADP-ribose) polymerase (PARP) (Fig. 5A). Only venetoclax or NaB treatment showed a little of cleaved PARP but most of pro-form still remained. The combined treatment with venetoclax and NaB caused a distinct band of the cleaved form and only a little pro-form remained. The results support the cytotoxic effect enhanced by the combination observed in cell cycle assay and trypan blue dye exclusion assay. Recently, various types of cell death such as necroptosis, autophagic cell death, pyroptosis and so on have been reported^14–18^. Thus, we investigated the cell death type caused the combination with venetoclax and NaB. We used Q-VD-OPh, a potent pan-caspase inhibitor^48–50^. As shown in Fig. 5B, the combination of venetoclax and NaB caused death for most of cells with a remaining of a small number of live cells, whereas single use of each reagent had mild effect on cell growth and cell death, which was the almost same with that of control. The Q-VD-OPh attenuated the cell death to the control level, while live cells were recovered to a half level. Observation under a microscope also demonstrated that most of dead cells in the combined treatment were disappeared by Q-VD-OPh and the sight was recovered to the same of control under a microscope (Fig. 5C). Cell cycle analysis with a flow cytometry confirmed the results. More than 50% of sub-G1 became to about 5% by Q-VD-OPh (Fig. 5D,E). The Q-VD-OPh also blocked the weak sub-G1 induced by only venetoclax treatment. These results indicated that most of cell death induced by the combined treatment was apoptosis.

### The combination of venetoclax with NaB did not affect on chronic myeloid leukemia K562 cells

**Figure 6:**
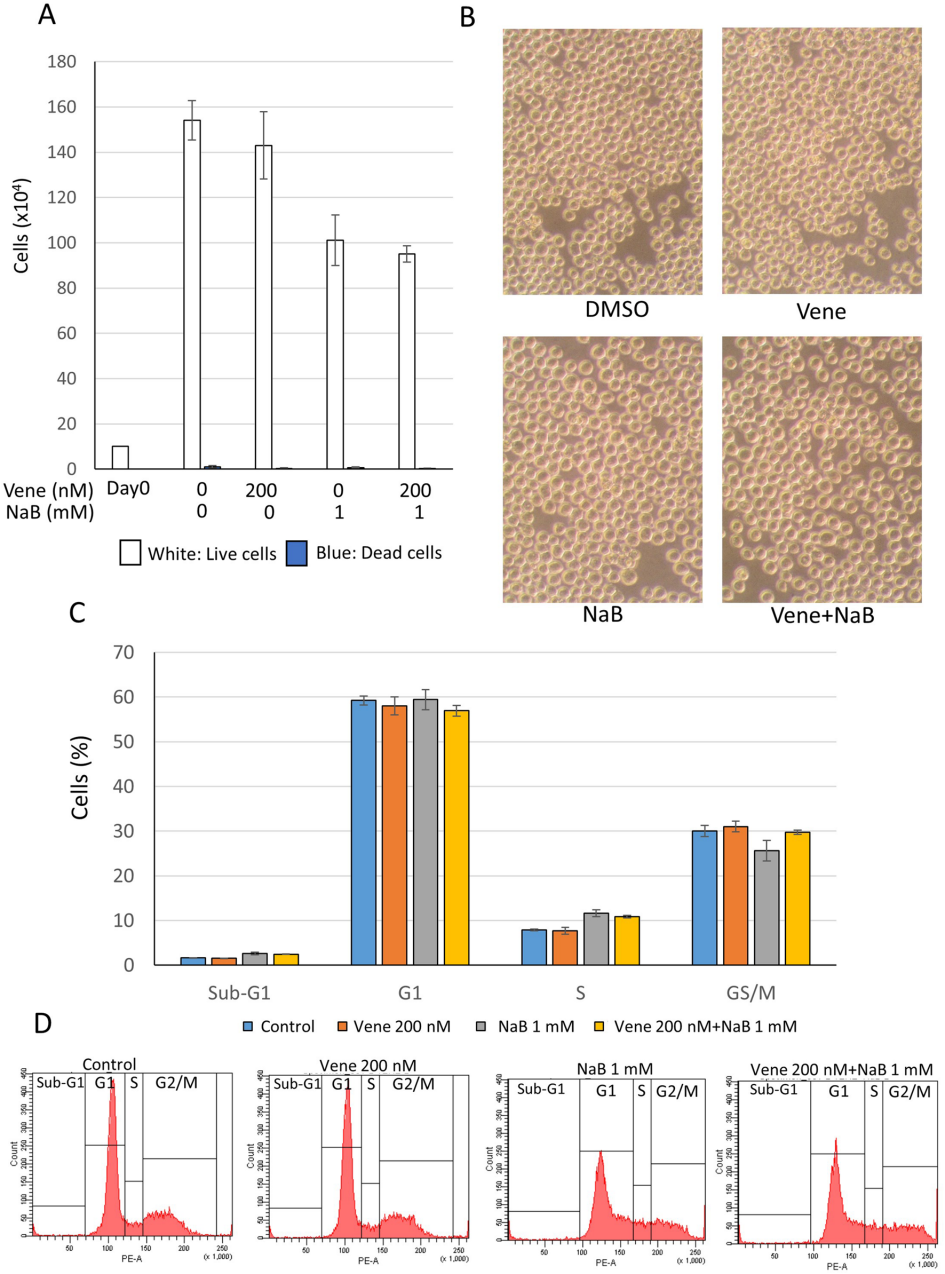
The combination of venetoclax with NaB did not affect on chronic myeloid leukemia K562 cells. K562 CML cell line was treated with the combination of venetoclax and NaB for 48 h. (**A**) Trypan blue dye exclusion assay to examine the rate of live or dead cells. (**B**) Cell morphologies were observed using a microscope. (**C**,**D**) Cell cycle analyses of cells stained with PI using flow cytometry. A bar graph with triplicate data (**C**) and representative histograms (**D**) were shown. The data was n = 3 ± S.D..

We examine the combined effect of venetoclax and NaB on another type of leukemia using K562 chronic myeloid leukemia (CML) cell line expressing BCR-ABL fusion protein by t(9;22) chromosome translocation. Surprisingly, trypan blue dye exclusion assay did not show any dead cells, while the number of live cells were moderately decreased (Fig. 6A). Venetoclax combined with NaB did not also change cell morphology in K562 cells (Fig. 6B). Cell cycle analysis using a flow cytometry demonstrated that not only a single agent but also the combination of venetoclax and NaB had no effect at all (Fig. 6C,D). These results indicated that the combined effect of venetoclax and NaB for cell death induction was a specific manner against AML cells.

## Discussion

In the present study, we demonstrated that venetoclax’s effect on AML cells were enhanced by the combination with NaB. In the concentration at which only venetoclax or NaB hardly induced cell death, the combination with two agents showed drastic cell death. The finding can lead to improve the restrictive effect of venetoclax against AML in clinical. Recently, AML resistant to venetoclax has been reported^51,52^. The combination with venetoclax and NaB may overcome the resistance. As a mechanism of the sensitization of AML cells to venetoclax by NaB, we demonstrated that NaB upregurated Bax and Bak. Venetoclax targets Bcl-2 which is an anti-apoptotic factor and inhibits apoptosis via interactions with Bax and Bak. Our results indicates that increased Bax and Bak by NaB easily stimulates mitochondria because of Bcl-2 inhibition by venetoclax, resulting in strong apoptosis induction. Moreover, as a mechanism of Bax and Bak upregulation by NaB, we found HDAC existence on Bax and Bak gene locus. We suggest that the expressions of Bax and Bak are usually suppressed by HDAC and NaB can cancel the inhibitions because NaB possesses a role in HDAC inhibitor. Hege Hurrish K et al. reported the effect of CUDC-907, a dual inhibitor of PI3K and HDAC, in combination with venetoclax in AML^53^ and Valdez BC et al. reported enhanced cytotoxicity of bisantrene, a topoisomerase-II inhibitor, when combined with venetoclax and panobinostat, an inhibitor of HDAC^54^. These reports support our suggestion.

Previously, we have reported that NaB enhances the anti-tumor effect of TRAIL in multiple types of cancer cells^44^. We also discovered that the RUNX1-ETO fusion protein attenuated TRAIL expression in t(8;21) AML cells, and that administration of recombinant TRAIL could induce apoptosis in AML cells and also reported that the effect of TRAIL could be enhanced by adding NaB at this time^45^. TRAIL is a promising anti-tumor agent that is undergoing clinical phase II and III trials^41–43^. Our results suggest that the triple combination of NaB, TRAIL, and venetoclax may further enhance the clinical efficacy of venetoclax.

Oral administration of NaB (5 g/kg) in mice produced butyrate concentration of plasma peaked at approximately 9 mM and NaB at 1.29 g/kg intravenous (i.v.) produced peak plasma butyrate concentration at 10.5–17.7 mM^55^. Tributyrin is a triglyceride composed of butyric acid and glycerol and used as a prodrug of butyrate^56^. Tributyrin is naturally occurred in butter, cheese and honey and it can be administrated through oral or intraperitoneal. Oral tributyrin administration (1 g/kg) increased the plasma butyrate level up to 2.4 mM in rat^57^. Another study reported that plasma butyrate concentration reached more than 1 mM by oral tributyrin administration (10.3 g/kg) and oral administration at the 7.8 g/kg caused plasma butyrate concentration at approximately 1 mM in mice^55^. We used 0.25–1 mM NaB in this study and the concentration could be reached easily in plasma by oral or i.v. administration of NaB or tributyrin in vivo. Therefore, our data suggest that butyric acid is a promising partner for the combination of venetoclax.

Some types of butyrate derivatives exist in human body and have been used as drugs. These derivatives such as phenylbutyrate approved for treatment of urea cycle disorders^58–60^, hydroxybutyrate, a ketone body produced during starvation^61–63^ and γ-aminobutyrate, a neurotransmitter^64^, also have properties as HDAC inhibitors. These butyrate derivatives may also play a role in a sensitizer of AML to venetoclax. Moreover, venetoclax was also conducted for phase III clinical trial of multiple myeloma^65^ and phase II clinical trials in B-cell lymphoma and breast cancer^66^. The combination with venetoclax and butyrate may be useful strategy for treatments of not only AML but also other malignant tumors.

Previous literatures showed that AML stem cells caused resistance to venetoclax by up-regulation of fatty acid oxidation (FAO) and inhibition of FAO restored the sensitivity to venetoclax^67–69^. Since NaB is a fatty acid, it may be effective that NaB is used together with an inhibitor of FAO in combination with venetoclax against AML stem cells.

In summary, we demonstrated here that NaB enhanced the venetoclax’s function of cell death induction, leading to a promising strategy for AML treatment.

## Supplementary Information

Supplementary Figure 1.

Supplementary Figure 2.

Supplementary Figure 3.

Supplementary Figure 4.

## Data Availability

For original data, please contact yoshida@koto.kpu-m.ac.jp.
