# Correction: Venetoclax efficacy on acute myeloid leukemia is enhanced by the combination with butyrate

**DOI:** 10.1038/s41598-025-09484-z

**Published:** 2025-07-11

**Authors:** Renshi Kawakatsu, Kenjiro Tadagaki, Kenta Yamasaki, Tatsushi Yoshida

**Affiliations:** Department of Biochemistry and Molecular Biology, Graduate School of Medical Science, Kyoto Prefectural University of Medicine, Kawaramachi-Hirokoji, Kamigyo-ku, Kyoto, 602-8566 Japan

Correction to: *Scientific Reports* 10.1038/s41598-024-55286-0, published online 29 February 2024

The original version of the Article contained an error in Fig. 5, where in Fig. 5C “Control” image was a duplication of the “NaB 1 mM” image.

The original Fig. 5 and accompanying legend appear below.

**Fig. 5:**
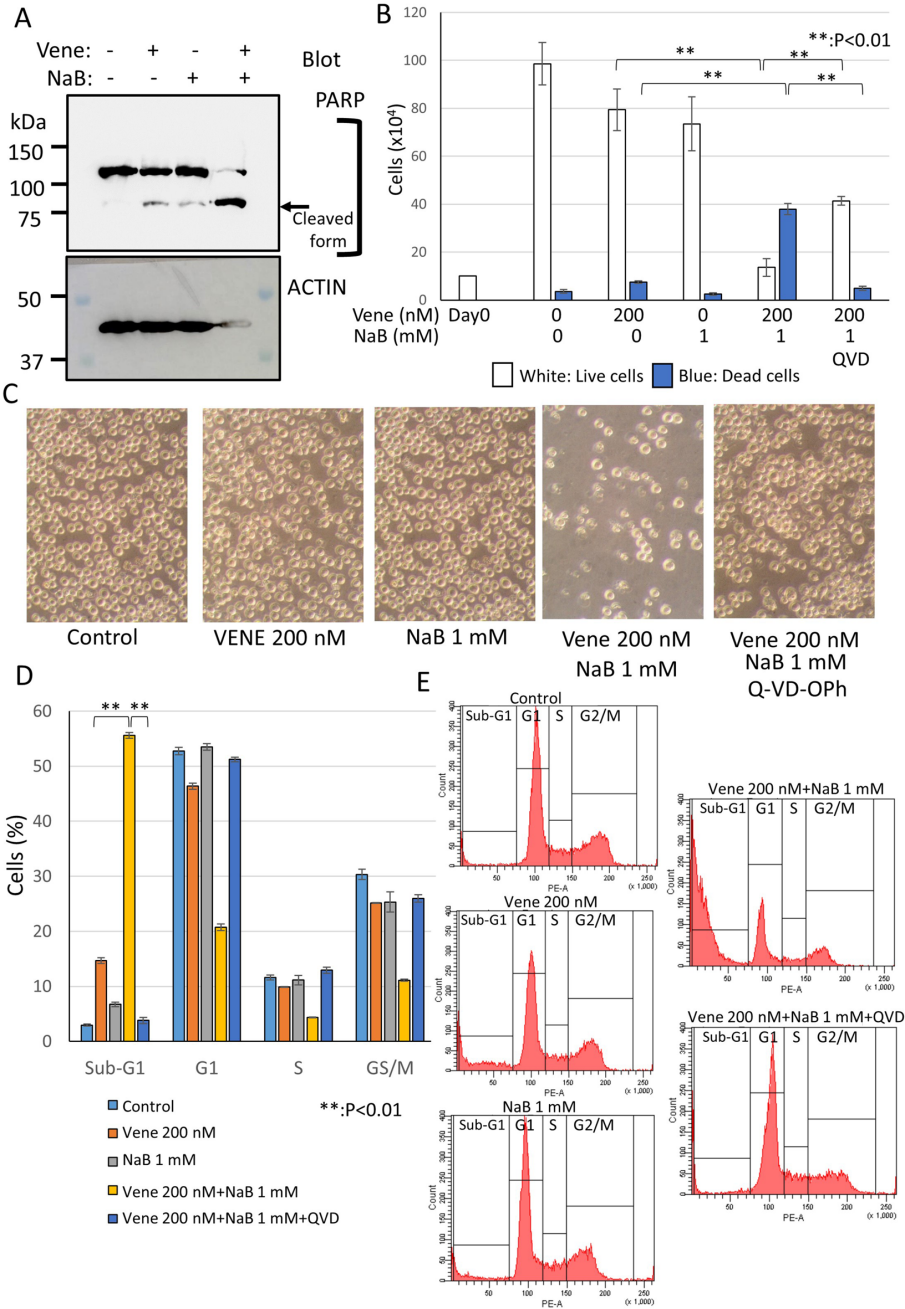
Caspase activation was induced by the combination of venetoclax and NaB. KG-1 was treated with venetoclax alone or the combination with venetoclax and NaB for 48 h. (**A**) Western blotting of PARP. Cell lysates were extracted and western blotting was performed. Upper panel; PARP, Lower panel; actin. Molecular weight marker was shown at the left side. The PARP-cleaved form generated by caspase activation was indicated by an arrow. Original blots are presented in Supplemental Fig. 3. Quantification of band intensity was shown in Supplemental Fig. 4. (**B**) Trypan blue dye exclusion assay to examine the rate of live or dead cells. (**C**) Cell morphologies were observed using a microscope. (**D**,**E**) Cell cycle analyses of cells stained with PI using a flow cytometry. A bar graph with triplicate data (**D**) and representative histograms (**E**) were shown. The data was n = 3 ± S.D.. Vene; venetoclax.

The original Article has been corrected.

