# Supplementary figures and images for: Venetoclax efficacy on acute myeloid leukemia is enhanced by the combination with butyrate
Source: Sci Rep. 2024 Feb 29;14:4975. doi: 10.1038/s41598-024-55286-0 (PMC10904797; doi:10.1038/s41598-024-55286-0)

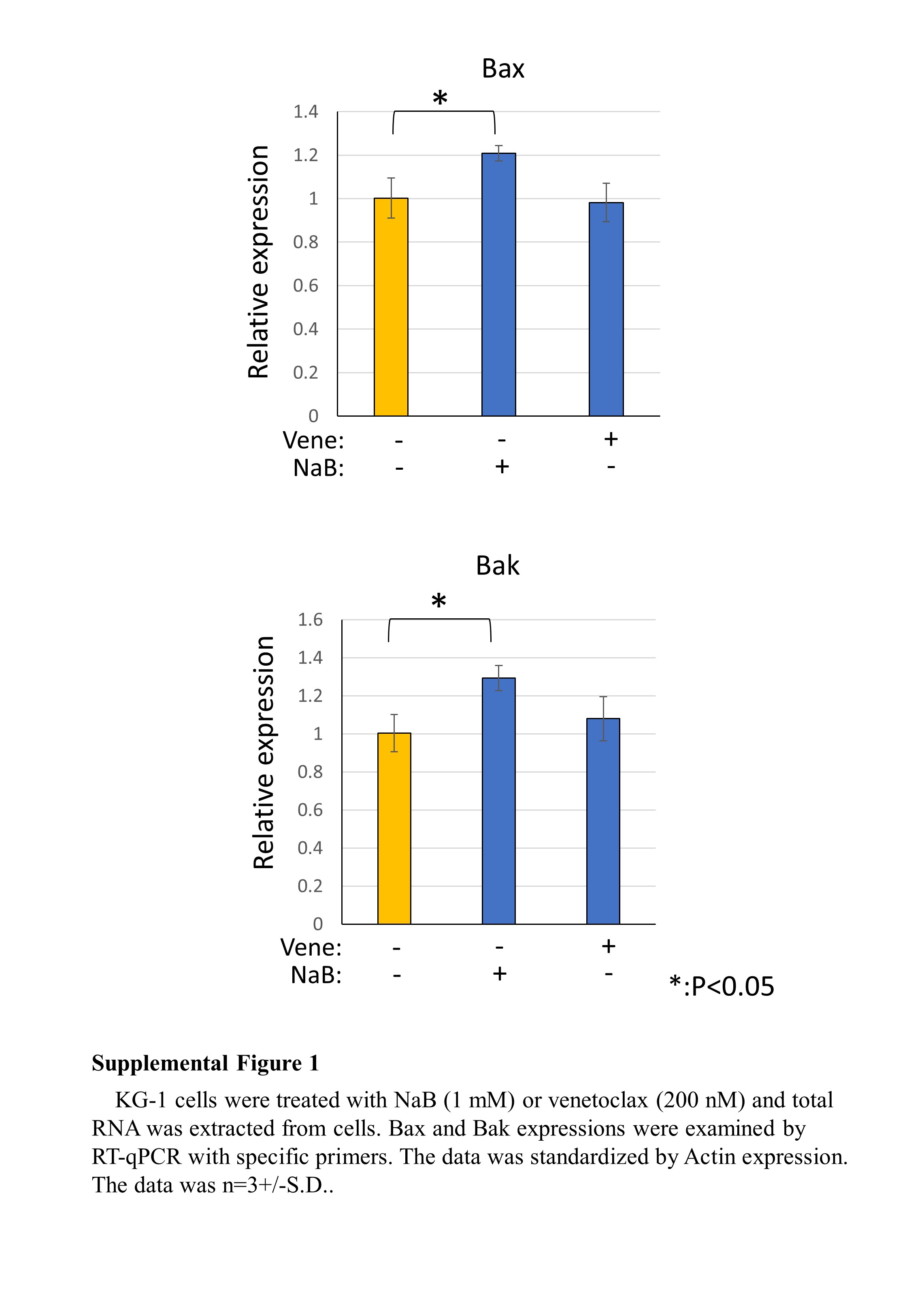

Supplement: Supplementary file 1 — Supplementary Figure 1. [file 41598_2024_55286_MOESM1_ESM.jpg]

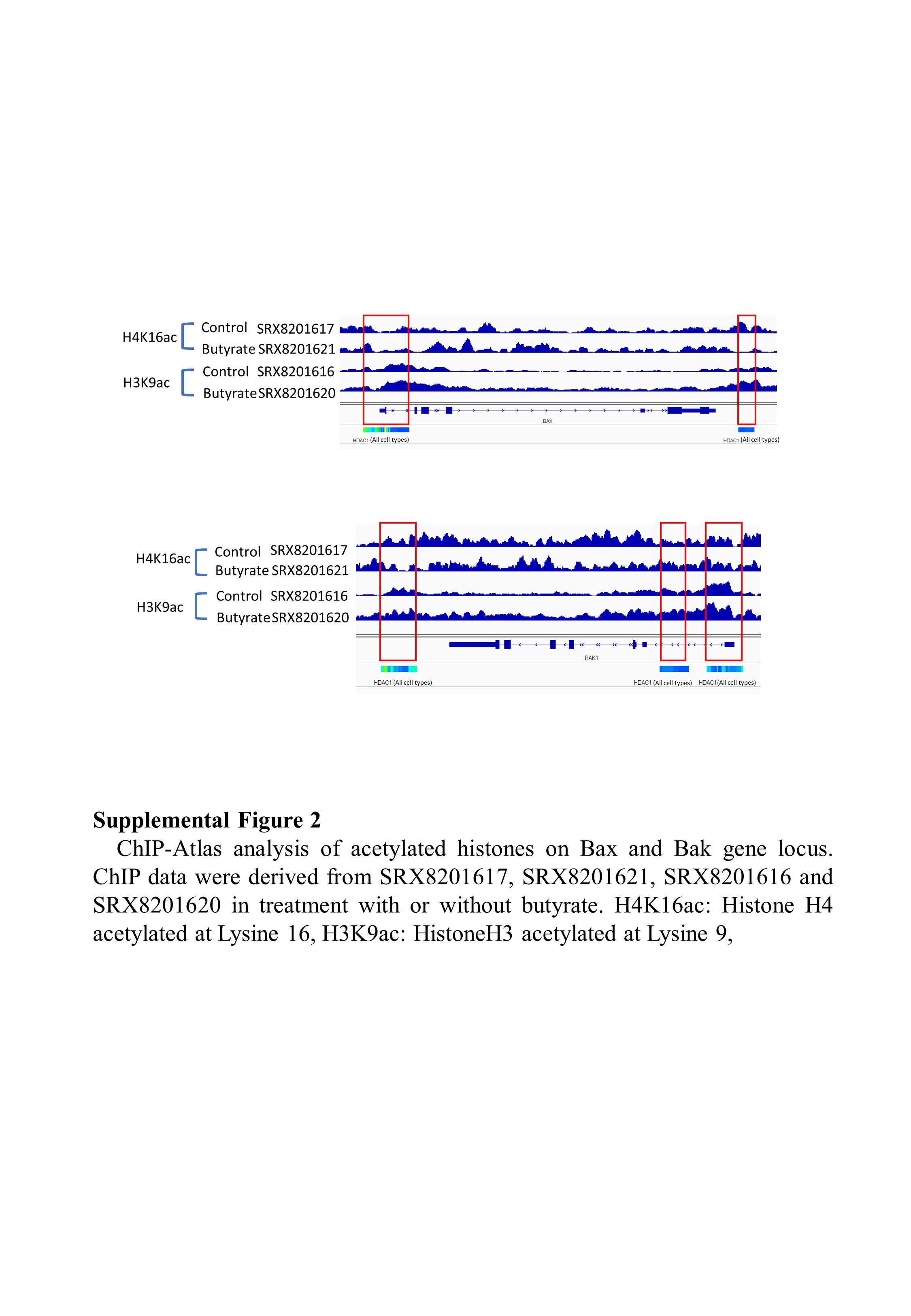

Supplement: Supplementary file 2 — Supplementary Figure 2. [file 41598_2024_55286_MOESM2_ESM.jpg]

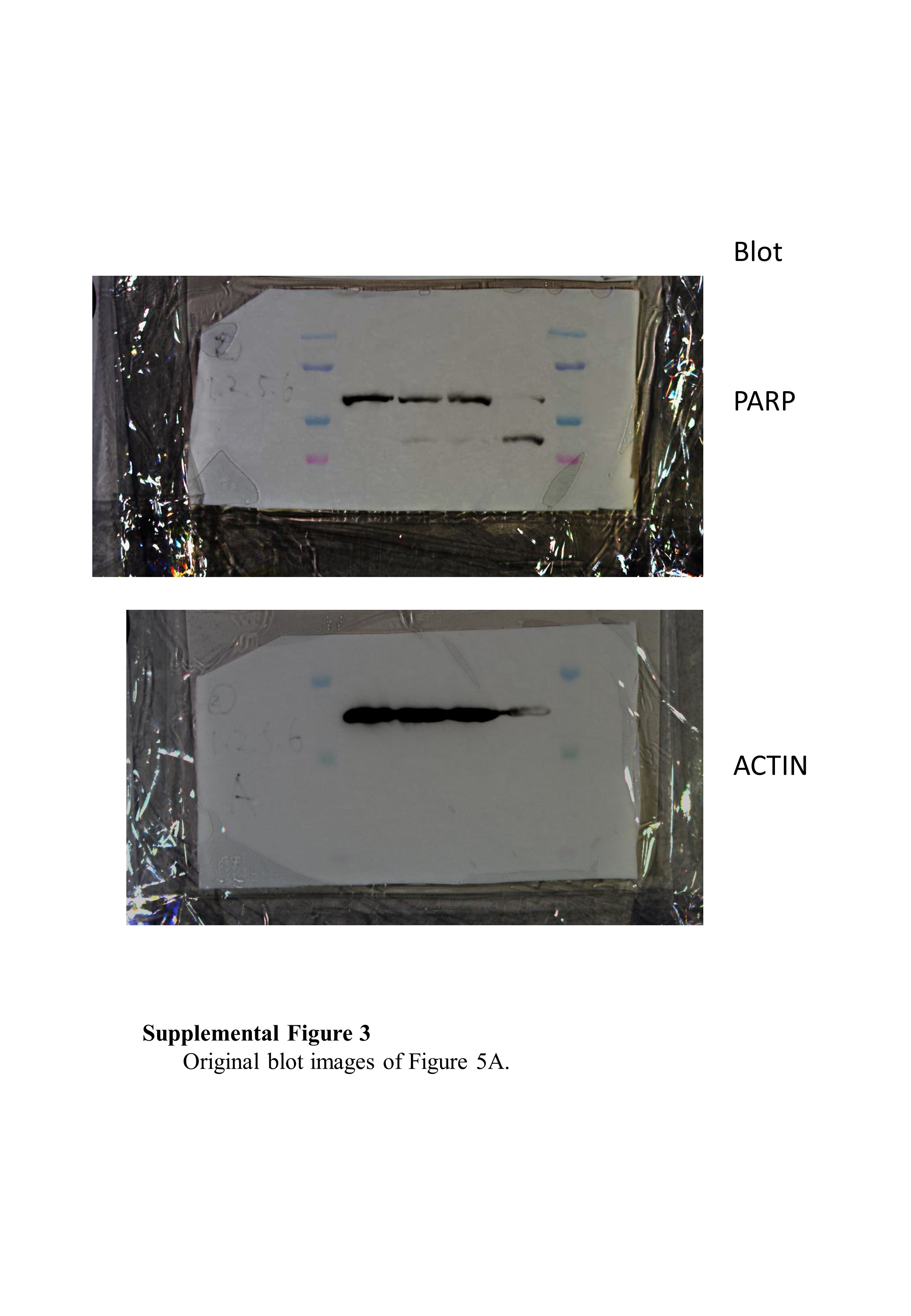

Supplement: Supplementary file 3 — Supplementary Figure 3. [file 41598_2024_55286_MOESM3_ESM.jpg]

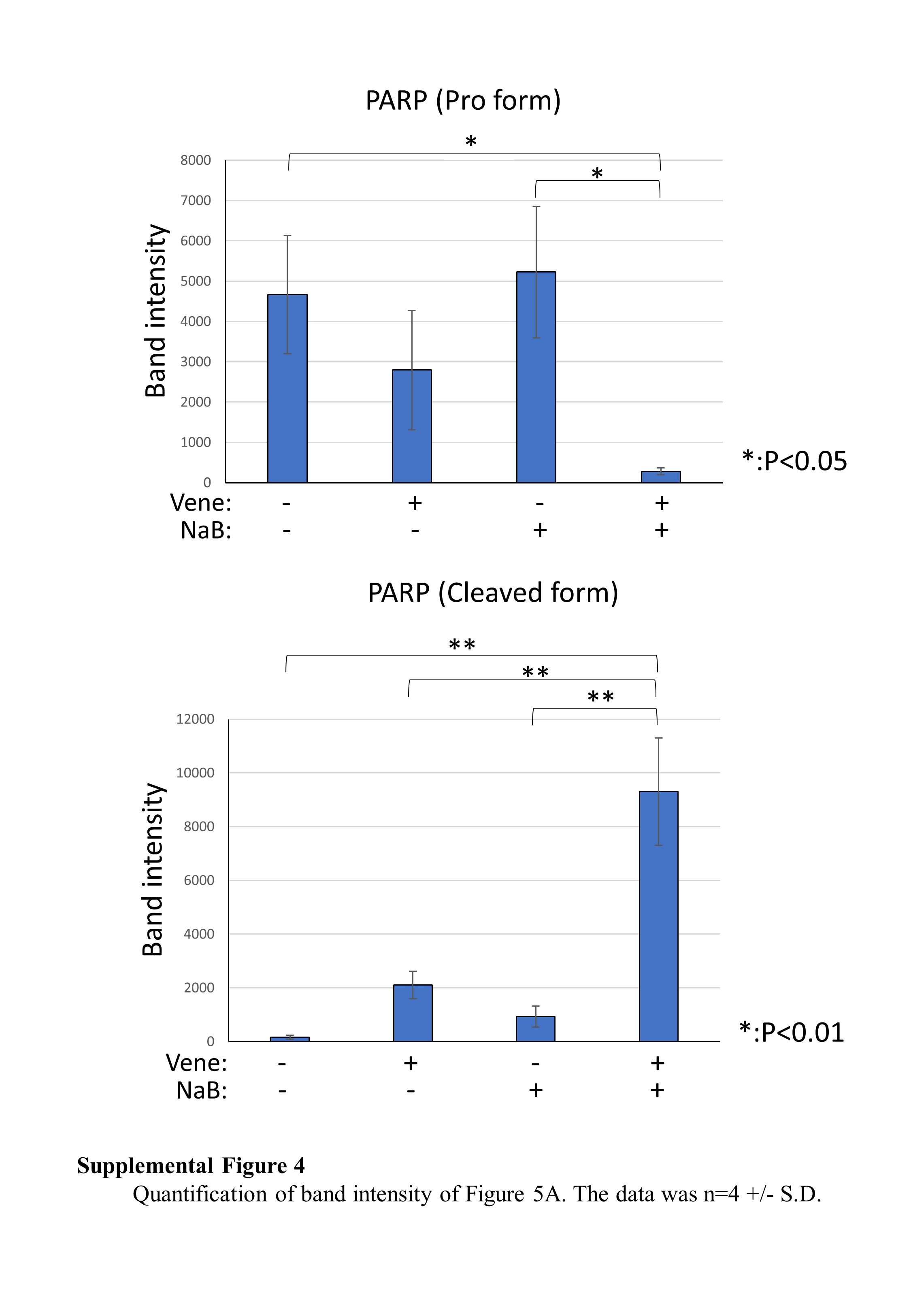

Supplement: Supplementary file 4 — Supplementary Figure 4. [file 41598_2024_55286_MOESM4_ESM.jpg]
